# Integrating Fundamental Movement Skills and Mathematics in Early Childhood: A Pilot Study

**DOI:** 10.3390/children11040457

**Published:** 2024-04-10

**Authors:** Catherine M. Capio, Sum Kwing Cheung, Serena S. W. Fung, Xinyun Hu

**Affiliations:** 1 Department of Physiotherapy, Hong Kong Metropolitan University, Kowloon, Hong Kong SAR, China; 2 Department of Early Childhood Education, The Education University of Hong Kong, Tai Po, Hong Kong SAR, China; sskcheung@eduhk.hk (S.K.C.); xinyunhu@eduhk.hk (X.H.); 3 School of Arts and Humanities, Tung Wah College, Kowloon, Hong Kong SAR, China; serenafung@twc.edu.hk

**Keywords:** fundamental movement skills, locomotor skills, object control skills, mathematics, physical activity, early childhood

## Abstract

This project involved a co-design process involving researchers and kindergarten teachers to produce learning activities that integrated fundamental movement skills (FMS) and mathematics. We piloted the co-designed activities (i.e., motor–math program) in a local kindergarten and examined the effects on FMS proficiency, mathematics skills, and accrued physical activity (PA). The participants comprised pupils (N = 39) from two matched kindergarten classes, in which we compared the motor–math program with typical mathematics lessons. All participants wore pedometers to measure their number of steps during class, one day per week. FMS proficiency (i.e., locomotor, object control) and mathematics skills (numeracy, geometry, math problem solving) were measured before and after implementation. Significant improvements in locomotor and object control skills were found only in the pilot group (*p* < 0.001); there were no differences in the changes in mathematics skills between the pilot and comparison groups. During implementation days, the participants in the pilot group accrued significantly greater step counts (*p* < 0.001) than those in the comparison group. Participating in the motor–math program appears to have benefits associated with improvements in FMS proficiency and accrued PA time, suggesting a promising potential for integrated activities as a means of PA promotion in kindergarten settings. Future work that examines the effects of the integration of movement with mathematics should consider randomization, greater sample size, and a longer intervention period.

## 1. Introduction

Physical activity (PA) participation has been consistently associated with a host of physical, psychosocial, developmental, cognitive, and mental health benefits among children from early years to adolescence [1,2]. Emerging evidence from longitudinal studies has also shown benefits in academic achievement [3]. Despite such associated benefits, insufficient PA continues to be a global health problem contributing to obesity and overall mortality [4]. This is evident in recent global estimates, which showed that less than one out of five adolescents aged 11 to 17 years accrue adequate PA [5]. We need to promote PA early in childhood, and schools are viable settings for such health promotion. Synthesized evidence has recently shown that programs in childcare and school settings can be effective in promoting PA [6,7].

PA patterns develop in early childhood and track across adolescence and adulthood [8]. Thus, PA promotion among young children is critically important. Strategies to promote PA in early childhood education (ECE) settings such as preschools and kindergartens have been increasing. A review of strategies in ECE settings showed that activities that focus on fundamental movement skills (FMS) development increase the intensity of PA while in school [6]. FMS consists of object control (i.e., throwing, catching, rolling), locomotor (i.e., crawling, running, jumping, hopping), and stability (i.e., stretching, rolling, balancing) skills [9]. These skills tend to emerge naturally in young children, but practice facilitates proficiency [10]. Motor development in early childhood is a key contributor to how children interact with their social and physical environments [11], and FMS are considered important enablers for children to engage in PA [12,13]. Thus, integrating FMS with mathematics can help children accrue sufficient PA indirectly through FMS proficiency and directly through an increased amount of active time in school [14,15].

Integrating FMS with literacy goals may allow ECE teachers to circumvent challenges associated with the growing emphasis on academic learning requirements. In Hong Kong, parents expect kindergartens to prepare their children for a successful academic future. Kindergarten teachers face challenges associated with academic priorities and consequent time constraints that limit their ability to promote both movement proficiency and PA [16]. Moreover, the ECE curriculum tends to be increasingly oriented towards academic preparation, impacting teachers’ prioritization for literacy goals [17,18].

In recent years, the integration of movement opportunities with teaching curricula has been recommended and examined in primary schools [19,20]. A recent review showed that physically active lessons, for example, yielded benefits in motor skills and academic performance among primary school students [14]. There has also been evidence that such integrated lessons enhanced students’ focus during class (i.e., time-on-task), which could contribute to academic learning processes [21]. There has been particular interest in integrating movement with mathematics because sensorimotor components are believed to transform mathematical concepts into tangible events or situations for young children, thereby facilitating effective learning [22]. For instance, a systematic review found 9 studies (out of 29 reviewed papers) that integrated movement with mathematics lessons [23]. The benefits of integrating movement with mathematics include improved geometry [24] and numeracy skills along with enhanced PA [25]. The growing evidence supporting the value of integrating movement with academic learning, however, has been mostly for primary school settings [14,23]. While specific frameworks have been proposed to integrate movement with learning in ECE [26], empirical work that integrates movement and PA with mathematics learning for pre-primary-school settings is still needed [15].

### Current Study

In this pilot study, we aimed to explore the potential benefits of integrating FMS with mathematics learning in a kindergarten setting. We expected that PA time might also increase as movement opportunities are integrated with learning activities, presenting a potentially viable PA promotion strategy. We considered that crucial to the delivery of integrated lessons is the commitment by ECE teachers. This can be achieved if the teachers are prepared to embrace the concept of learning through movements [27] and their perspectives are considered in the design of the activities [28]. Thus, the program of activities was co-designed by kindergarten teachers and the research team. We refer to this as the motor–math program. Engaging a co-design processes also addresses the issue that most research tends to test programs designed by researchers and implemented in school contexts with minimal to no involvement of teachers during the planning stage [6], despite teacher-led interventions yielding more benefits for children [29].

We piloted the co-designed motor–math program in one kindergarten class, while a matched class served as a comparison. Our primary research objective was to explore the following outcomes following participation in the motor–math program: (i) FMS proficiency–, i.e., locomotor and object control skills, (ii) mathematics skills–, i.e., numeracy, geometry skills, and problem-solving skills, and (iii) PA time in the kindergarten. With a pre-post study design, we hypothesized that all participants would display significant improvements in FMS proficiency and mathematics skills, but the improvements would be greater for participants in the pilot group. We also hypothesized that participants in the pilot group would accrue greater PA time compared to those in the comparison group during the implementation of the motor–math modules. Finally, we aimed to verify whether the kindergarten teachers delivered the motor–math program according to how it was co-designed (i.e., fidelity).

The project was affected by a pandemic-related school suspension period, which commenced immediately following our planned pre-tests and lasted for four months. Due to the prolonged school suspension, we repeated our pre-tests of FMS proficiency and mathematics skills upon school resumption. While it was unplanned, we measured FMS proficiency and mathematics skills before and after a school suspension period. Thus, we added a secondary research objective to explore the effects of school suspension on the FMS proficiency and mathematics skills of young children.

## 2. Materials and Methods

### 2.1. Co-Design of Motor–Math Activities

This pilot study is situated in a local kindergarten that follows the government-mandated kindergarten curriculum in Hong Kong [30]. In the local system, there are three kindergarten levels (i.e., K1, K2, K3) with pupils aged three to five years. Three educators (i.e., one vice principal and two class teachers) and two researchers with backgrounds in motor development and skills acquisition (i.e., pediatric physiotherapist, outdoor education practitioner) co-designed activities for the K2 level. Two other experienced kindergarten teachers reviewed the activities and recommended revisions to refine the design. All the involved teachers had >5 years of experience in their current roles, while the principal and researchers had >10 years of experience in their areas of expertise. The activities were designed for the development and learning areas of (i) FMS and (ii) early childhood mathematics. The learning objectives followed the curriculum of the local kindergarten in these two learning areas. Eight activities were co-designed and revised through three iterative cycles. Activity sheets with step-by-step instructions, equipment requirements, and illustrations were produced for kindergarten teachers to use. One short video clip was also created for each activity to provide an alternative medium for teachers’ reference. All media were originally developed in Chinese for local users and subsequently translated to English for later dissemination to non-Chinese speakers.

### 2.2. Pilot Implementation

The institutional ethics review committee reviewed and approved all the study procedures (Ref. no. 2019-2020-0123). We initially planned a 10-week implementation consisting of a pre-test (week 1), an activity implementation (weeks 2–9), and a post-test (week 10). In the week following the pre-test, a pandemic-related school suspension (including kindergartens) was imposed in the city. After the schools resumed in-person classes, we recommenced the project implementation but deemed the four-month period that had lapsed to be significant to child development. As such, we administered a second round of pre-test (week 1), which was followed by a shortened activity implementation (weeks 2–6) and finally an immediate post-test (week 7). We shortened the activity implementation due to the curricular and time constraints in the kindergarten following the suspension period.

#### 2.2.1. Participants

Two classes of a local kindergarten were deemed comparable based on participants’ age (i.e., same K2 level) and sex distribution. Family socioeconomic status was also deemed comparable because the kindergarten caters to children from an area with relatively homogeneous socioeconomic characteristics. Both classes follow the same curricular programming, consistent with the local government standards. Using a concealed envelope procedure, each class was randomly allocated to either the motor–math (MM) or comparison (CM) group. A total of 41 children aged 46 to 69 months (mean age at pre-suspension 52.85 SD 4.80 months; 19 males) were eligible to participate and provided parental consent. However, due to absences during measurement days, complete data were recorded for only 20 children in the MM group (mean age at pre-suspension 53.65 SD 4.18 months; 7 males) and 19 children in the CM group (mean age at pre-suspension 52.00 SD 5.36 months; 10 males). No significant differences were found in age (t(37) = −1.07, *p* = 0.29) or sex distribution (χ^2^(1) = 1.23, *p* = 0.27) between the MM and CM groups. All the participants were typically developing children and had no diagnosed neurodevelopmental, medical, or orthopedic condition that is contraindicated to moderate intensity physical activity; none required special educational needs support.

#### 2.2.2. Instruments

FMS proficiency was measured using the Test of Gross Motor Development—Third Edition (TGMD-3) [31]. The TGMD-3 has two sub-tests—locomotor and object control skills—which consist of seven and six skills, respectively. High levels of internal consistency, reliability, and validity have been established for the TGMD-3 [32,33]. It has also been used in studies involving young children in Hong Kong [34]. Trained research support staff administered the tests following standard procedures that consisted of a demonstration by the tester, followed by two test trials for the participant. All tests were recorded on video and were scored post hoc by two trained raters who were blind to the participants’ group allocation. Excellent inter-rater reliability was established based on a random sample of 10 videos (r = 0.98, *p* < 0.001). Each of two trials was scored based on the presence (score = 1) or absence (score = 0) of observable performance criteria. Higher scores represent greater gross motor skill proficiency, where the highest possible raw score for locomotor skills was 46, and for object control skills was 54. Raw scores were recorded for analysis, which is suitable for our objective of examining change over time [35]. It is also consistent with the approach of previous studies that involved samples other than those from North American populations, which form the basis of normative data [34,36].

Mathematics skills were measured using three components of the KeyMath 3 Diagnostic Assessment (KeyMath 3): numeration, geometry, and problem solving [37]. KeyMath 3 consists of ten components that test a spectrum of mathematics concepts and skills from kindergarten to ninth grade and has been used to assess pre-primary school children in Hong Kong [38]. The selected components are untimed verbal-response and picture-based measures. Numeration measures the skills of number comparison, counting, and simple calculation; geometry measures understanding of two- and three-dimensional shapes, spatial relationships and reasoning, coordinates, and symmetry; and applied problem solving measures the application of mathematical concepts to real-life contexts. These three components comprise 120 items that are arranged in order of difficulty. Trained research support staff administered the tests, which began with the easiest item in each component and progressed with increasing difficulty until the participant failed to give the correct answer for five consecutive items. Each correct item was scored 1, where the highest possible score for each component was 49 for numeration, 36 for geometry, and 35 for applied problem solving. We used the component raw scores for our analysis because other components that are needed to convert to a standard score were excluded, and the normative data were not representative of our study population [39].

PA levels were measured using pedometers (Digi-Walker CW700, Yamax Co., Kumamoto, Japan). The Digi-Walker has been validated for measuring physical activity among preschool children [40]. Pedometers measure the number of steps that the user takes and are widely considered a suitable tool for objective measurement of ambulatory physical activity in children [41]. More recently, pedometers have been recommended as a suitable tool for physical activity surveillance in early childhood [42]. The participants wore pedometers on their right hip, which were sealed to prevent reactivity [41]. The number of step counts was recorded, supported by evidence-based guidelines suggesting that step counts are preferable over energy expenditure estimates in children [43,44].

#### 2.2.3. Procedures

The integrated motor–math program was implemented in the MM group during the time allocated for mathematics, while typical mathematics learning activities (i.e., without the movement components) were implemented in the CM group. The motor–math activities are shown in Appendix A, along with the relevant FMS learning objectives. We note that the same learning objectives were targeted in the typical mathematics activities for the CM group. For both the MM and CM groups, the teachers also targeted FMS learning objectives during the time allocated for play as mandated by the local kindergarten curriculum.

The two teachers in charge of the MM group were orientated to the co-designed activities and presented with the reference materials (i.e., activity sheets, video clips). The motor–math program consists of eight activities that were designed for an eight-week implementation. The teachers were guided by the following parameters: (i) one activity per week, (ii) three class sessions for each activity, and (iii) a minimum of 15 min per session. Due to the shortened implementation period (i.e., five weeks), the teachers of the MM group were allowed to select which specific activity they would conduct in each week. They also decided on which day (i.e., three out of five days) and time (i.e., at least 15 min out of three hours) within the class schedule they would implement the chosen activity. We determined that this approach afforded the teachers some amount of flexibility as they implemented the program during the pandemic period.

During the five-week implementation period, the participants wore pedometers clipped to their waistband over the right hip during a three-hour class once weekly—on a day when motor–math activities were conducted. TGMD-3 and KeyMath 3 were administered three times—prior to school suspension (pre-suspension), after school suspension and prior to implementation (post-suspension/pre-test), and immediately following implementation (post-test). The administrators for all tests were blind to the participants’ group allocation.

To verify fidelity, the lead teacher of the MM group was asked to record details on an online platform (i.e., Qualtrics) once weekly: (i) chosen activity for the week, (ii) number of sessions of activity implementation in that week, (iii) duration of each session, (iv) modifications of the activity (if any), and (v) difficulties (if any) and strategies to manage them. A member of the research staff also conducted class observations once per week and documented similar implementation aspects as follows: (i) chosen activity for the week, (ii) duration of the session, (iii) consistency of the activity with the designed task, and (iv) modifications (if any).

#### 2.2.4. Data Analysis

Effect sizes were calculated using partial eta squared, and statistical significance was set at *p* < 0.05. We first performed Pearson’s product-moment correlation test to confirm that the two FMS subskills (i.e., locomotor, object control) are correlated; a similar test was performed for the three mathematics skills components (i.e., numeracy, geometry, problem solving). To examine change following the program, we performed 2 (group: MM, CM) × 2 (time) repeated measures multivariate analysis of covariance (MANCOVA) separately on FMS and mathematics skills while controlling for pre-test scores as covariates. We controlled for pre-test scores in the MANCOVA to account for differences prior to the implementation. Levene’s and Box’s M tests verified that assumptions of homogeneity of variance and covariance were not violated in the analysis for both FMS and mathematics skills (all *p* > 0.05). Significant effects were followed up by univariate tests, pairwise comparisons, and separate paired samples (i.e., pre-post scores) *t*-tests with Bonferroni corrections for the MM and CM groups.

To compare the amount of accrued PA during class hours, a 2 (group: MM, CM) × 5 (time) repeated measures analysis of variance (ANOVA) was performed on the pedometer data. Mauchly’s test showed that the assumption of sphericity was violated, χ^2^(9) = 33.26, *p* < 0.001. Hence, the degrees of freedom were corrected using the Greenhouse–Geisser estimates (ε = 0.76). Significant main effects were followed up by pairwise comparisons, and a significant interaction effect was followed up by separate repeated measures ANOVAs per group.

To examine the effect of school suspension on FMS and mathematics skills, we performed 2 (group: MM, CM) × 2 (time) repeated measures MANCOVA while controlling for pre-suspension scores on the full sample. Levene’s and Box’s M tests verified that the assumptions of homogeneity of variance and covariance were not violated (all *p* > 0.05).

Data from the teacher’s reports and class observations were analyzed descriptively to understand the implementation of the motor–math activities by teachers.

## 3. Results

### 3.1. FMS Proficiency

The participants’ mean scores (95% confidence interval) in locomotor and object control skills across the three measurement points are summarized in Table 1. Multivariate tests showed a significant main effect of time (F(2, 34) = 21.53, *p* < 0.001, ηp^2^ = 0.56) and a significant interaction between group and time (F(2, 34) = 14.39, *p* < 0.001, ηp^2^ = 0.46) on FMS proficiency.

Univariate tests showed a significant main effect of time on locomotor skills (F(1, 35) = 34.50, *p* < 0.001, ηp^2^ = 0.50) and a significant interaction between group and time (F(1, 35) = 12.36, *p* = 0.001, ηp^2^ = 0.26). The change in score over time was influenced by the pre-test score, as evident in the significant interaction between pre-test score and time (F(1, 35) = 18.19, *p* < 0.001, ηp^2^ = 0.34). Paired samples *t*-tests showed that the MM group displayed significant improvement in locomotor skills scores over time (t(19) = −7.02, *p* < 0.001, d = −1.57). The improvement of the CM group over time was not significant (t(18) = −1.69, *p* = 0.11, d = −0.39).

Univariate tests showed a significant main effect of time on object control skills (F(1, 35) = 12.73, *p* = 0.001, ηp^2^ = 0.27) and a significant interaction between group and time (F(1, 35) = 19.42, *p* < 0.001, ηp^2^ = 0.36). The change in score over time was influenced by the pre-test score, as evident in the significant interaction between pre-test score and time (F(1, 35) = 13.35, *p* = 0.001, ηp^2^ = 0.28). Paired samples *t*-tests showed that the MM group displayed significant improvements in object control skills scores over time (t(19) = −5.71, *p* < 0.001, d = −1.28). The improvement of the CM group over time was not significant (t(18) = −1.00, *p* = 0.33, d = −0.23).

These findings suggest that participants of the MM group gained greater improvements in locomotor and object control skills compared to the CM group participants. The participants’ proficiency prior to implementation influenced their improvements.

### 3.2. Mathematics Skills

The participants’ mean scores (95% confidence interval) in numeracy, geometry, and problem solving across the three measurement points are summarized in Table 1. Multivariate tests showed that the main effect of time was not significant (F(3, 32) = 1.624, *p* = 0.20, ηp^2^ = 0.13), and the interaction between group and time was not significant (F(3, 32) = 0.16, *p* = 0.92, ηp^2^ = 0.02) for mathematics skills.

Univariate tests showed that for numeracy scores, the pre-test score had a significant interaction with time (F(1, 34) = 12.28, *p* = 0.001, ηp^2^ = 0.27). The main effect of time (F(1, 34) = 2.07, *p* = 0.16, ηp^2^ = 0.06) and the interaction between group and time (F(1, 34) = 0.05, *p* = 0.83, ηp^2^ = 0.01) were not significant.

Univariate tests for geometry scores showed that the pre-test score had a significant interaction with time (F(1, 34) = 20.70, *p* < 0.001, ηp^2^ = 0.38). The main effect of time (F(1, 34) = 2.18, *p* = 0.15, ηp^2^ = 0.06) and the interaction between group and time (F(1, 34) = 0.05, *p* = 0.82, ηp^2^ = 0.01) were not significant.

Univariate tests for problem-solving scores showed that the pre-test score had a significant interaction with time (F(1, 34) = 8.97, *p* = 0.005, ηp^2^ = 0.21). There was a significant main effect of time (F(1, 34) = 4.27, *p* = 0.04, ηp^2^ = 0.11), but the interaction between group and time (F(1, 34) = 0.23, *p* = 0.64, ηp^2^ = 0.01) was not significant.

The findings suggest that only problem-solving skills improved following the implementation period, but there were no differences between the MM and CM group participants. The participants’ scores prior to implementation influenced the changes following implementation.

### 3.3. Physical Activity

**Figure 1:**
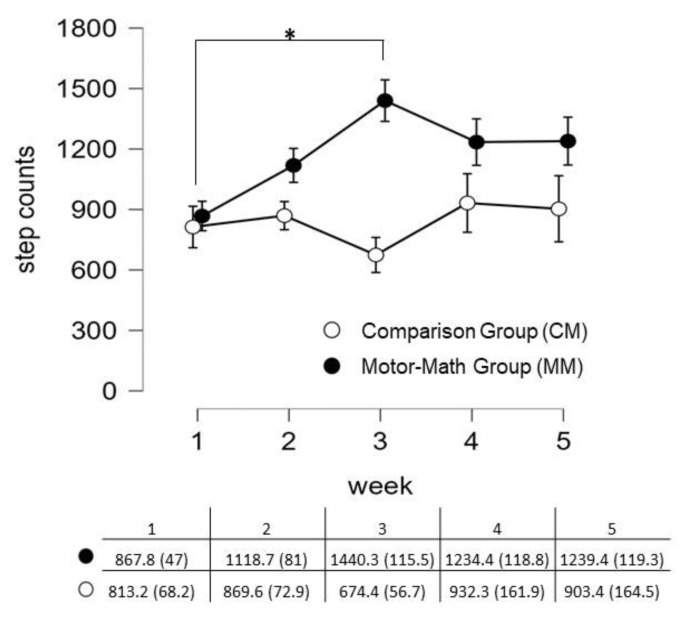
Mean (SE) step counts recorded during class, once weekly over the five-week implementation period of the motor–math (MM) and comparison (CM) group. * Statistically significant difference.

There was a significant main effect of group on the participants’ number of steps (F(1, 47) = 22.89, *p* < 0.001, ηp^2^ = 0.33), but the main effect of time was not significant (F(4, 188) = 1.67, *p* = 0.174, ηp^2^ = 0.03). Post hoc pairwise comparison showed that the MM group accrued a greater number of steps than the CM group (*p* < 0.001). There was a significant interaction between group and time (F(4, 188) = 2.83, *p* = 0.04, ηp^2^ = 0.06). Follow-up separate repeated measures ANOVAs showed that a significant effect of time was apparent only for the MM group (F(4, 96) = 4.36, *p* = 0.003, ηp^2^ = 0.15) but not for the CM group (F(4, 92) = 0.73, *p* = 0.57, ηp^2^ = 0.03). Pairwise comparisons for the MM group showed that the number of steps increased after time 1, with a significant change found between time 1 and time 3 (*p* = 0.001); the number of steps remained high through time 4 and time 5, as changes were not significant as illustrated in Figure 1. These findings suggest that participants of the MM group generally accrued more physical activity time than the CM group participants. The MM group’s mean step accounts also had lower standard errors at the last two weeks of implementation, suggesting that the group had become more homogeneous towards the end of the intervention period (see Figure 1).

### 3.4. Fidelity and Teachers’ Feedback

The records made by the teachers of the MM group revealed that all eight activities were used over the five-week implementation period. Three activity sessions were conducted each week (a total of 15 sessions), where seven activities were implemented for two sessions each, and one activity was implemented once. Each session lasted at least 20 min per session, meeting the minimum recommendation of 15 min per session. The teachers also reported that six out of the eight activities were implemented exactly as designed without any modifications. They were deemed easy to set up and deliver in their typical classroom settings. There were two activities that were deemed relatively difficult due to challenging task components for children at the K2 (i.e., 4 to 5 years old) level—i.e., reading written cues, knowing their own birthdays, and remembering actions linked to each day of the week. In these activities, the cognitive components were modified to reduce the difficulty level (i.e., verbal instead of written cues, modified recall requirements including birthdays), but the motor components were retained. The overall feedback from the teachers is that the motor–math activities can be implemented in their local setting, and they thought that the children learned the mathematics concepts of shapes, days in a week, and numeracy while being active.

The observation records of the research staff confirmed that each observed session lasted for at least 20 min and that the co-designed activities were implemented as intended. Modification was observed in one activity, where children needed to know their birthdays. It was noted that the teacher assisted the children in determining their birthday when they did not know it themselves. The motor (i.e., balancing) and numeracy (i.e., counting in groups) aspects of the activity were retained. All other observed activities were consistent with the co-designed plan.

### 3.5. Effects of School Suspension

Multivariate tests showed a significant main effect of time (F(2, 35) = 15.33, *p* < 0.001, ηp^2^ = 0.47) on FMS proficiency. Univariate tests revealed a significant main effect of time on locomotor skills (F(1, 36) = 28.54, *p* < 0.001, ηp^2^ = 0.44) and a significant interaction between time and pre-suspension scores (F(1, 36) = 11.41, *p* = 0.002, ηp^2^ = 0.24). These findings suggest that locomotor skills improved during the school suspension period, and their pre-suspension scores influenced change over time.

Univariate tests showed a significant main effect of time on object control skills (F(1, 36) = 17.43, *p* = 0.001, ηp^2^ = 0.33) and a significant interaction between time and pre-suspension scores (F(1, 36) = 6.30, *p* = 0.02, ηp^2^ = 0.15). These findings suggest that object control skills improved during the period of school suspension, and the change over time was influenced by the pre-suspension score.

Multivariate tests showed a significant main effect of time (F(3, 33) = 7.85, *p* < 0.001, ηp^2^ = 0.42) on mathematics skills. Univariate tests for numeracy scores showed a significant main effect of time (F(1, 35) = 21.11, *p* < 0.001, ηp^2^ = 0.38) and a nearly significant interaction of time with pre-suspension score (F(1, 35) = 4.11, *p* = 0.05, ηp^2^ = 0.11). These findings suggest that numeracy improved during the period of school suspension and that the pre-suspension score possibly influenced change over time.

Univariate tests showed that the main effect of time on geometry scores was not significant (F(1, 35) = 2.08, *p* = 0.16, ηp^2^= 0.06), but there was a significant interaction between time and pre-suspension score (F(1, 35) = 24.02, *p* < 0.001, ηp^2^ = 0.41). These findings suggest that while the improvements in geometry skills were not significant over the period of school suspension, pre-suspension scores influenced change over time.

The univariate test for problem-solving skills showed a significant main effect of time (F(1, 35) = 7.25, *p* = 0.01, ηp^2^ = 0.17) and a significant interaction of time with the pre-suspension score (F(1, 35) = 4.57, *p* = 0.04, ηp^2^ = 0.12). These findings suggest that problem-solving skills improved during the school suspension period and that the pre-suspension scores influenced change over time. The participants’ scores on the five variables prior to school suspension, post-suspension/pre-test, and post-test are summarised in Table 1.

## 4. Discussion

In this study, we co-designed integrated activities for early childhood learning objectives in FMS and mathematics. Our fidelity check confirmed that the activities were implemented according to the design but with a reduced dosage as a consequence of the shortened implementation period. Each activity was conducted no more than twice, while the original plan was to conduct each three times.

We note that the motor–math program was designed to be consistent with the learning objectives of the local ECE curriculum. Thus, all the participants from both the pilot and comparison groups participated in learning activities that aimed to improve FMS proficiency and early childhood mathematics skills. The only difference in the participants’ experiences was that the time allocated for mathematics was typically sedentary for the comparison group and integrated with movement for the pilot group. As such, the pilot group was exposed to greater time allocated for FMS practice, the byproduct of which is greater PA time. We hypothesized that our pilot would have beneficial effects on FMS proficiency, mathematics skills, and time spent engaging in PA.

### 4.1. Enabling FMS Proficiency

Our findings showed potential benefits associated with FMS development in kindergarten children. We suggest that this is an important finding for ECE educators to consider because FMS emerge and develop in early childhood, forming the foundations for more complex skills that children would perform in primary school and beyond [9]. By enabling exploration of the environment and interaction with peers, FMS contribute to cognitive and socioemotional development [45,46]. ECE curricula, such as those in Hong Kong, target FMS proficiency within the area of physical development and health [30]. Health benefits are associated with FMS proficiency because they are known to be significant enablers of children’s PA engagement [47]. Thus, integrating movement with learning activities contributes direct benefits to PA time, and indirect benefits by way of improved FMS proficiency.

### 4.2. Increasing Physical Activity in Educational Settings

Consistent with our expectation of direct benefits to PA, the children in our pilot group accrued greater PA during class hours over the implementation period compared to those in the comparison group. Similar to studies in primary school contexts [48,49], our findings show that integrating movement with learning areas in educational settings can help children meet the recommended amount of PA. The World Health Organization recommends that children below five years old should accumulate 180 min of PA, of which 60 min is moderate to vigorous intensity [50]. The health authorities in Hong Kong adopted these recommendations but applied them to children aged three to six years, corresponding to the age of those in kindergarten [51]. We recognize that for children to meet these guidelines, activity patterns outside of ECE settings are important contributors and parent/family factors are significant determinants [52]. Nevertheless, our pilot supports the idea that there is potential for ECE settings to contribute to helping children meet PA guidelines [7] despite ECE programs lasting only three hours per day. Further work following this pilot study could explore the extent to which ECE settings deliver this contribution to PA promotion.

### 4.3. Supporting Mathematics Skills

It has been suggested that integrating movement with mathematics could be advantageous in terms of sensorimotor experiences and embodied cognition [53], and improved self-regulation [54], which contributes to mathematics learning [55,56]. Our findings did not support these expectations as we found no differences in the changes in mathematics skills between the pilot and comparison groups. The mathematics learning objectives across all children were the same during the implementation period, and all participants displayed improvements. Despite the integration of movement with mathematics, we did not find any of the expected advantages.

A systematic review of integrated mathematics lessons in primary schools showed that significant improvements in mathematics were found in those studies that implemented programs for longer durations—i.e., at least one year [15]. Shorter interventions, such as those in this pilot study, are likely of inadequate dosage to yield benefits in mathematics skills. Moreover, the pilot group effectively had greater time allocated to practicing FMS relative to the comparison group while the time allocated to mathematics content was generally similar between groups. This could potentially explain the evident lack of advantages in mathematics skills associated with the integrated activities. Future work that builds on this pilot, therefore, should consider (1) a longer intervention period to determine any potential effects on mathematics skills in young children and (2) enhancing the practice opportunities for mathematics content. From another perspective, the lack of difference in the mathematics outcomes between the pilot and comparison groups also indicates that the FMS and PA benefits did not come at the expense of mathematics learning. In societies such as Hong Kong, where academic learning is highly prioritized [16], these findings could show parents and educators that movement and PA in ECE settings will not have any detrimental effect on the highly prioritized academic outcomes.

### 4.4. Insights on Child Development

Due to pandemic-related constraints, we administered measurements before and after a period of school suspension. While not in the original study design, we assessed the impact of school suspension on the FMS proficiency and mathematics skills of young children. During the four-month period of school suspension, the conditions were presumably similar for the children given the pervasive pandemic-related social restrictions in the city. Hence, we found similar patterns of change in skills. FMS proficiency improved and this reflects developmental change [9], which tends to support the idea that FMS naturally develop even in the absence of interventions. We note, however, that the implementation period was much shorter (i.e., five weeks) than the school suspension period (i.e., four months), but we also found significant improvements in FMS, but only for those in the pilot group. Thus, we suggest that our findings support the notion that training and practice are important for FMS to be efficiently mastered [10].

The children also gained improvements in numeracy and problem-solving skills during the period of school suspension. A large-scale survey of parents of preschool children in Hong Kong during the pandemic revealed that parents were actively involved in implementing learning activities, including mathematics, at home during periods of school suspension [57]. We speculate that the participants’ parents effectively facilitated learning activities in numeracy and problem solving at home, hence the observed improvements.

Across FMS sub-skills and mathematics skill components, the level of proficiency prior to the suspension period contributed to the changes in the children’s skills during the suspension period. Similarly, the pre-test scores contributed to the changes in the children’s skills following the intervention period. These findings suggest that individual differences remain significant contributors to early childhood learning and development despite interventions. In other words, those who have higher skill levels are more likely to display greater improvements over time than those who have lower skill levels. Nevertheless, interventions such as the integrated activities in this pilot study could facilitate greater improvements in movement proficiency. However, we highlight the importance of accounting for individual differences when designing interventions. Previous research emphasized that the design of motor skill interventions needs to account for individual differences and their underlying factors [58].

### 4.5. Strengths and Weaknesses of the Study

While most investigations that integrated movement with mathematics have focused on primary school children, this study contributes evidence for younger children. Moreover, studies of intervention programs in education settings rarely involved frontline teachers in the planning process. One strength of this current study is the engagement of ECE educators in co-designing the integrated activities, thereby producing a program with the perspectives of teachers themselves [28]. Nevertheless, we acknowledge that the functional links between the motor and mathematics components could be improved from a strengthened interdisciplinary perspective, such that a program revision is warranted prior to further trials.

The study was interrupted due to COVID-19-pandemic-related school suspensions in Hong Kong. The interruption compelled us to collect data from three time points (instead of two), which allowed us to address a secondary research objective. However, this also led to a shortened implementation period, which likely limited the likelihood of gaining benefits to mathematics skills [15]. Based on this pilot study, future work could implement integrated activities over longer periods (e.g., one school year) and could explore whether a dose–response relationship exists between movement and mathematics skills.

FMS is conceptualized to consist of locomotor, object control, and stability skills [9]. However, we did not measure stability in this pilot, and future work could consider additional batteries to assess FMS comprehensively.

As a pilot, our sample size was small, and random allocation was only at the class level. In addition, the involvement of only one class each for the pilot and comparison groups may not rule out the possibility that any improvements in outcomes can be accounted for by the teacher. Nevertheless, our findings will inform further research with a more robust design (e.g., randomized controlled trial) that could potentially generate clearer evidence. While our fidelity check also suggests that the integrated program is acceptable to teachers, a comprehensive formative evaluation alongside a robust trial is recommended to better understand the parameters of program delivery.

## 5. Conclusions

This pilot study showed that integrating FMS and mathematics might be beneficial for kindergarten-aged children in terms of FMS development and PA time. There was no difference in mathematics skills, likely due to the short duration of the intervention, and practice opportunities were enhanced for FMS but not for mathematics skills. In addition, we found that young children displayed improvements in both FMS proficiency and mathematics skills during a period of pandemic-related school suspension, highlighting the role of parents in facilitating development and learning in periods of school disruptions. The current findings inform the design of further research that will aim to integrate movement with mathematics in ECE contexts and evaluate their impact on children’s motor development, PA participation, and mathematics learning.

## Figures and Tables

**Table 1:** Mean (95% CI) of the gross motor and mathematics scores of the motor–math (MM) and comparison (CM) groups over the three measurement points.

Variables	Motor–Math Group (*n* = 20)	Comparison Group (*n* = 19)
**Gross motor skills**		
*Locomotor*		
Time 1	15.70 (11.88–19.52)	13.11 (9.97–16.24)
Time 2	25.10 (21.79–28.41)	25.16 (20.94–29.38)
Time 3	34.05 (31.45–36.65)	28.05 (24.52–31.59)
*Object control*		
Time 1	12.05 (9.82–14.28)	9.47 (7.26–11.69)
Time 2	21.10 (17.92–24.28)	20.68 (16.99–24.38)
Time 3	30.35 (26.61–34.10)	22.05 (19.18–24.92)
**Mathematics**		
*Numeracy*		
Time 1	3.90 (2.99–4.81)	3.21 (2.52–3.90)
Time 2	5.55 (4.42–6.68)	5.21 (3.85–6.57)
Time 3	5.65 (4.45–6.85)	5.05 (3.85–6.26)
*Geometry*		
Time 1	4.35 (3.13–5.57)	3.74 (2.21–5.27)
Time 2	4.55 (2.78–6.32)	3.84 (2.68–5.00)
Time 3	6.00 (4.28–7.72)	5.42 (4.20–6.64)
*Problem solving*		
Time 1	2.90 (2.21–3.59)	2.00 (1.30–2.70)
Time 2	3.60 (2.65–4.55)	2.79 (2.12–3.46)
Time 3	3.70 (2.87–4.53)	3.00 (2.18–3.82)

## Data Availability

The data that support the findings of this study are available on request from the corresponding author, C.M.C. The data are not publicly available due to specifications in the agreement with the partner kindergarten at the time of the study implementation.

## Appendix A

Summary of the activities in the co-designed motor–math program.

**Table A1:** Learning objectives and activities of the Motor–Math program.

Early Childhood Mathematics Objective	FMS Objective	Activity Description
To learn the shapes oval and circle	To push and roll a rubber wheel while walking/running	Floor tapes (8 m in length) are set on the ground to form two shapes (circle and oval). The pupils will be divided into two groups, corresponding to the circle and oval shape. In the first round, pupils will walk on the tape following the shape assigned to them, and then swap shapes. In a subsequent round, pupils will push a rubber wheel while walking on the tape following the shape assigned to them, and then swap shapes.
To understand spatial positions (i.e., top, middle, bottom)	To throw with one hand (i.e., overhand, underhand)	A throwing target that consists of three pictures will be prepared: clouds on top, mountains, in the middle, and a lake at the bottom. Pupils will take turns throwing towards the pictures in the target. The teacher will help the students identify the spatial location of each picture that they hit by throwing. In a subsequent round, the pictures will be removed, and only top, middle, and bottom spaces will be left on the target.
To learn the ascending order of 1 to 5	To side-step To cross-step forward	Several sets of footprints with numbers 1 to 5 printed on them will be set on the ground in ascending order. Pupils will take turns following the footprints in ascending sequence, by side-stepping, and then cross-stepping forward, while also counting aloud.
To learn the combinations and decomposition of 5 and 6	To run and dodge others	Pupils will stand in random positions in a designated open space. The teacher will give a variety of instructions related to the combination of 5 or 6—e.g., “Form groups of 3 girls and 2 boys”. The children will then run around the open space to form groups.
To compare fast and slow	To run/walk To side-step To cross-step	Prepare music with contrasting rhythms—fast and slow. The teacher will play each type of music for each form of locomotion. Pupils will move following the rhythm, one round for each type of music and each form of locomotion
To remember the seven days in a week	To balance while holding an object To push/roll a rubber wheel while walking/running	The teacher will assign an activity to represent each day of the week (e.g., Monday: balance with feet together while holding a ball overhead; Tuesday: roll a rubber wheel while walking; Wednesday: balance with feet together while holding a ball in front at shoulder level). Pupils will demonstrate the assigned movements/activities for each day in sequence (i.e., Monday to Sunday). In a subsequent round, the teacher will call out a random day of the week, and students will demonstrate the corresponding movement/activity for that day.
To learn about map grids	To balance while holding an object To throw with one hand	Set a print of the map of Hong Kong on the floor (minimum size 3 m × 3 m). Superimpose a grid with 6 squares horizontally and 6 squares vertically on the map. A physical activity is assigned to each grid (e.g., balance while holding a hoop overhead; throw a beanbag overhead; throw a beanbag underhand). Prepare a large cube that will function as a dice with numbers 1 to 6. Each pupil will throw the dice twice to generate one number each for the horizontal and vertical grid. They will walk to the corresponding grid and perform the designated physical activity on that grid.
To count 20 by using 2 in a group, 5 in a group	To balance while holding an object	Floor tapes are set on the ground to form two lines—one line will have three marked graduations to represent a start spot, number 1 and number 2; another line will have six graduations to represent a start spot and numbers 1 through 6 Pupils will be divided into two groups, who will take turns stepping on each of the lines. From the start spot, a pupil counts aloud (e.g., 1, 2) as they step into a marker and maintain their balance with feet together; they will repeat until they are able to count 20 steps in total. In a subsequent round, the pupils will be given an object (e.g., basketball, hoops) to carry while stepping into a marker and balancing their feet together.

Note: The same learning objectives were targeted in the typical (non-integrated) activities of the comparison group.
